# Identification and validation of CCL5 as a key gene in HIV infection and pulmonary arterial hypertension

**DOI:** 10.3389/fcvm.2024.1417701

**Published:** 2024-07-25

**Authors:** Mengyue Yang, Wen Bi, Zhijie Zhang

**Affiliations:** ^1^ Department of Cardiology, Huazhong University of Science and Technology Union Shenzhen Hospital, The 6th Affiliated Hospital of Shenzhen University Health Science Center, Shenzhen, China; ^2^ Department of Sports Medicine, Huazhong University of Science and Technology Union Shenzhen Hospital, The 6th Affiliated Hospital of Shenzhen University Health Science Center, Shenzhen, China

**Keywords:** HIV infection, PAH, DEGs, CCL5, molecular docking

## Abstract

**Background:**

The relationship between human immunodeficiency virus (HIV) infection and pulmonary arterial hypertension (PAH) has garnered significant scrutiny. Individuals with HIV infection have a higher risk of developing PAH. However, the specific mechanism of HIV-associated PAH remains unclear. Our study aims at investigating the shared biomarkers in HIV infection and PAH and predicting the potential therapeutic target for HIV-associated PAH.

**Methods:**

Data for HIV infection and PAH were downloaded from Gene Expression Omnibus (GEO) database. Differentially expressed genes (DEGs) analysis was performed to detect shared genes in HIV infection and PAH. Enrichment analysis was conducted to identify the function of common DEGs. Protein-protein interaction (PPI) analysis was used to detect key genes. These crucial genes were subsequently verified by RT-qPCR. Finally, candidate drugs were identified by using the Drug Signatures Database (DSigDB).

**Results:**

Nineteen common DEGs were identified in HIV infection and PAH. Enrichment analysis exhibited that the functions of these genes were mainly enriched in inflammatory responses, mainly including cellular immunity and interaction between viral proteins and cytokines. By constructing PPI networks, we identified the key gene CC-type chemokine ligand 5 (CCL5), and we verified that CCL5 was highly expressed in hypoxia induced human pulmonary artery endothelial cells (hPAECs) and human pulmonary artery smooth muscle cells (hPASMCs). In addition, we predicted 10 potential drugs targeting CCL5 by Autodock Vina.

**Conclusion:**

This study revealed that CCL5 might be a common biomarker of HIV infection and PAH and provided a new therapeutic target for HIV-associated PAH. However, further clinical validation is still indispensable.

## 1 Introduction

HIV is a retrovirus that includes two types: HIV-1 and HIV-2 (1). HIV infection is a serious public health problem that has caused approximately 40 million deaths worldwide (2). Acquired immunodeficiency syndrome (AIDS) is the final outcome of HIV infection, and people diagnosed with AIDS often die from serious infections or cancer because of their highly weakened immune systems. In recent years, with the application of antiviral drugs, the life span of HIV infected people has been greatly extended (3). Thus, the chronic complications of HIV infection have attracted more attention. Previous studies indicated that HIV infection was linked to a higher risk of cardiovascular disease, which may be connected to persistent inflammation (4). Among them, HIV-associated PAH has received great attention because of its poor prognosis. A previous study indicated that PAH was a long-term complication of HIV infection, and individuals with HIV-associated PAH had a worse survival rate compared with HIV-positive patients without PAH (5). The prevalence of PAH is 0.5% in HIV-positive individuals, which is much greater than that in HIV-negative individuals (6).

As a subtype of pulmonary hypertension, pulmonary arterial hypertension is distinguished by pulmonary arterial remodeling. Right heart failure is the usual cause of death for PAH patients, causing a huge disease burden (7). Studies elucidating the mechanism of the relationship between HIV infection and PAH are very limited. DNA damage response and chronic inflammation may contribute to the development of HIV-associated PAH (8, 9). Besides, previous studies showed that increased levels of asymmetric dimethylarginine (10) and smooth muscle cell proliferation (11) also played key roles in HIV-associated PAH. In order to detect novel therapeutic targets for HIV-associated PAH, studying the common mechanism between PAH and HIV infection is very crucial.

Nowadays, we are able to better understand diseases owing to advancements in sequencing technology and bioinformatics analysis. In this study, we identified the common biomarkers between HIV infection and PAH and predicted potential drugs for HIV-associated PAH.

## 2 Materials and methods

### 2.1 Transcriptome data

**Figure 1:**
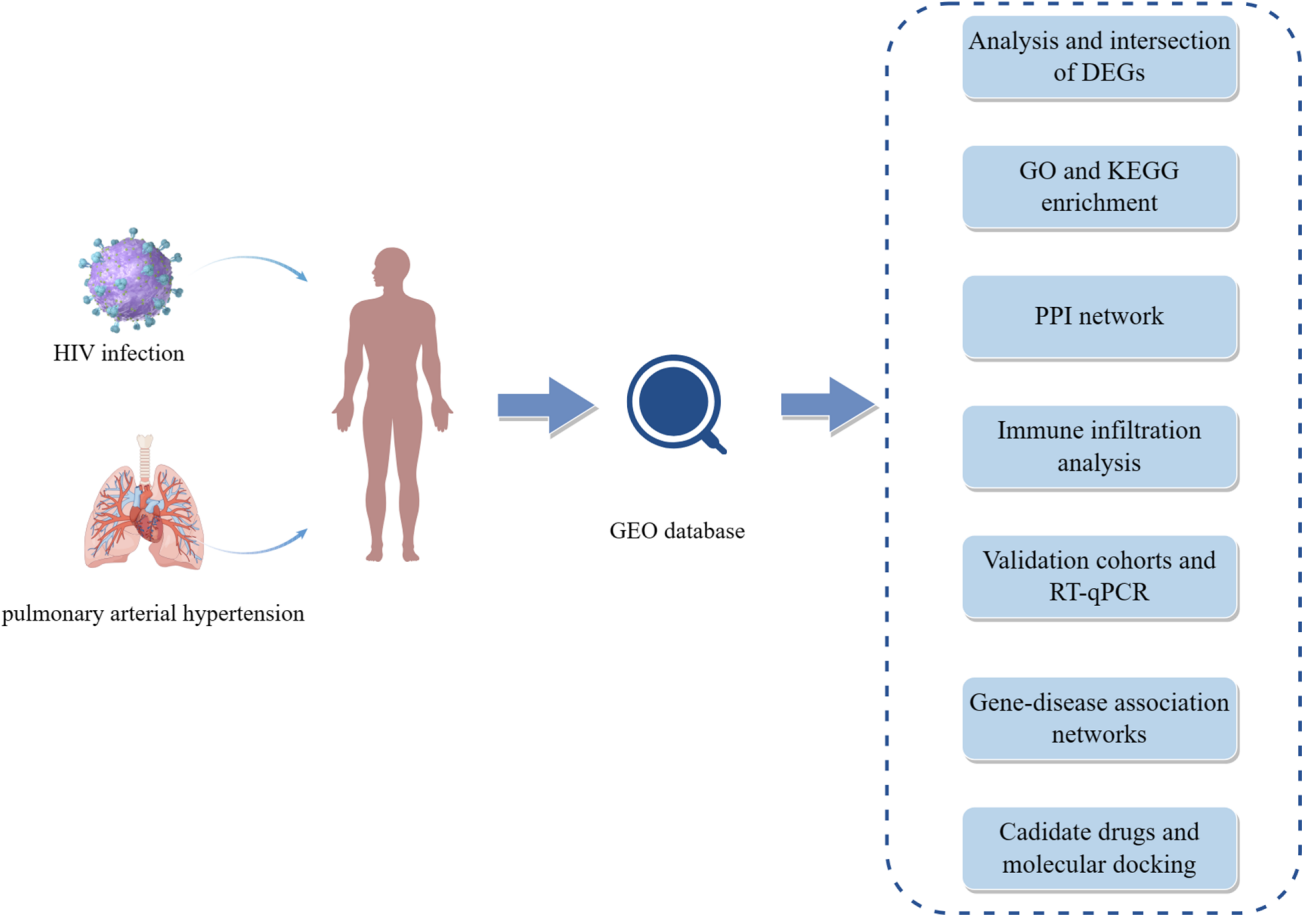
The workflow of this study. HIV, human immunodeficiency virus; GEO, gene expression omnibus; DEGs, differentially expressed genes; GO, gene ontology; KEGG, Kyoto encyclopedia of genes and genomes; PPI, protein-protein interaction; RT-qPCR, quantitative real-time polymerase chain reaction.

Datasets on HIV infection and pulmonary arterial hypertension were obtained from the GEO database (https://www.ncbi.nlm.nih.gov/geo/) (12). For discovery cohorts, dataset GSE37250 contained 274 HIV-positive and 263 HIV-negative whole blood samples (13), and dataset GSE117261 included lung tissue samples from 58 PAH patients and 25 controls (14). For validation cohorts, dataset GSE30310 involved 48 HIV-positive and 19 HIV-negative peripheral blood mononuclear cell samples (15), and dataset GSE53408 comprised lung tissue samples from 12 PAH patients and 11 healthy controls (16). All the data were derived from human species. Details of these datasets can be found in Supplementary Table S1. Figure 1 (by Figdraw) exhibits the workflow of this study.

### 2.2 Identification of common DEGs

DEGs analysis was performed by using “limma” package. For dataset GSE37250, the cutoff criteria was |Log2 fold change| >0.1 and adjusted *P*-value <0.001 (17). For dataset GSE117261, the cutoff criteria was |Log2 fold change| >0.585 and adjusted *P*-value <0.05 (18). The above results were visualized by heatmaps and volcano plots. The common DEGs of the two diseases were obtained by using “venn” package (19).

### 2.3 Functional enrichment analysis

Gene Ontology (GO) and Kyoto Encyclopedia of Genes and Genomes (KEGG) enrichment analyses are commonly used to find the biological function of gene sets (20, 21). We conducted function enrichment analysis of common DEGs by using “ClusterProfiler” package. *P*-value <0.05 was considered significant.

### 2.4 PPI network

Using the STRING database (version 12.0; https://cn.string-db.org/) (22), we built a PPI network of common DEGs. The minimum required interaction score was 0.150, and unconnected nodes were hidden. Besides, the PPI network was visualized by Cytoscape (version 3.10.1) (23). Subsequently, Molecular Complex Detection (MCODE) (24) was used to identify functional modules, and the default parameters were set as follows: degree cutoff = 2, node score cutoff = 0.2, K-core = 2, and max depth = 100. Finally, the top ten genes were identified by CytoHubba plugin according to Degree algorithm (25).

### 2.5 Immune cells infiltration analysis

Single sample gene set enrichment analysis (ssGSEA) algorithm was utilized to calculate enrichment scores between distinct groups by “GSVA” R package (26, 27). Next, the abundance of 28 types of immune cells in each group was visualized. Plus, we explored the linkage between hub genes and immune cells by Spearman's rank correlation analysis.

### 2.6 Cell culture

hPAECs were purchased from ScienCell (Shanghai, China, Cat. No. 3100). hPASMCs were obtained from Procell (Wuhan, China, Cat. No. CP-H243). They were cultured in endothelial cell medium (ScienCell, Cat. No. 1001) and smooth muscle cell medium (Procell, Cat. No. CM-H243), respectively, at 37°C with 5% CO_2_. Hypoxia induced hPAECs and hPASMCs were incubated with 2% O_2_, 5% CO_2_ and 93% N_2_ at 37°C for 24 h in anoxic incubators.

### 2.7 Quantitative real-time PCR

RNA was extracted from cells by Trizol reagent (Invitrogen, USA, Cat. No. 15596026). Next, the purity of the RNA was determined using NanoDrop 2000 (Thermo, USA). Then, the PrimeScript®RT Reagent Kit with gDNA Eraser (Takara, Japan, Cat. No. RR047A) was used for reverse transcription in T100 Thermal Cycler (Biorad, USA). Finally, qPCR was executed employing the SYBR®Premix Ex TaqII kit (Takara, Japan, Cat. No. RR820A) in qPCR instrument (Applied Biosystems 7500, USA). The primer sequences are displayed in Supplementary Table S2.

### 2.8 Gene-disease association analysis

DisGeNET is one of the largest collections of genes and variants linked to human disease (28). We constructed gene-disease association networks filtering by disease class infections and cardiovascular diseases, respectively. The above process was completed in DisGeNET Cytoscape App (version 7.3.0).

### 2.9 Validation of the docking protocol and molecular docking

The top ten candidate drugs with the best statistical significance were obtained from DSigDB database (29) by Enrichr (https://maayanlab.cloud/Enrichr/) (30) (Supplementary Figure S1). Small molecule drug structures were obtained from PubChem (https://pubchem.ncbi.nlm.nih.gov/). Structures of CCL5 (PDB code: 5DNF) were accessed from the PDB database (https://www.rcsb.org/). Autodock Vina software (31) and Pymol (32) were used for validation of the docking protocol and molecular docking. Firstly, the co-crystalline ligand (beta-D-glucopyranose) of CCL5 was extracted and saved. Then the co-crystalline ligand was re-docked with CCL5. The root mean square deviation (RMSD) between the conformation of the redocked ligands and the conformation of the original crystal structure was calculated to validate the docking protocol (33). Finally, the molecular docking process between ten drugs and CCL5 was carried out.

### 2.10 Statistical analysis

R software (v. 4.3.0) and GraphPad Prism (v.9.5.1) were employed in statistical analysis. Correlation between CCL5 and immune cells was determined by Spearman correlation analysis. *P*-value <0.05 was considered statistically significant.

## 3 Results

### 3.1 Identification of DEGs in HIV infection and PAH

**Figure 2:**
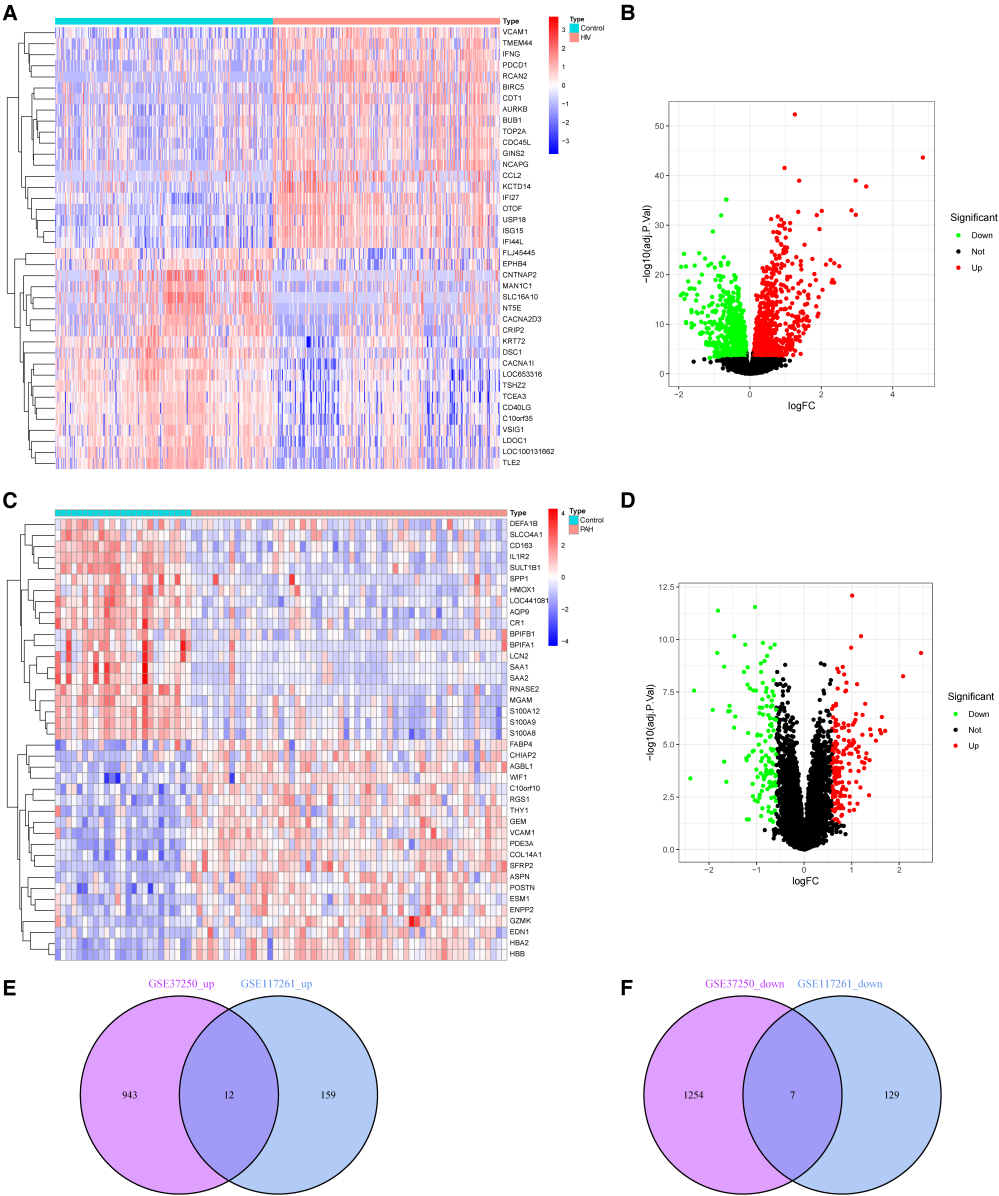
DEGs in HIV infection and PAH group. (**A,B**) Heatmap and volcano plot of DEGs in HIV infection. (**C,D**) Heatmap and volcano plot of DEGs in PAH. (**E,F**) The intersection of DEGs between HIV infection and PAH. DEGs, differentially expressed genes; HIV, human immunodeficiency virus; PAH, pulmonary arterial hypertension.

2216 DEGs and 307 DEGs were identified in datasets GSE37250 and GSE117261, respectively (Supplementary Table S3). Figures 2A,B showed the DEGs of HIV infection, and Figures 2C,D showed the DEGs of PAH. Further, the common DEGs were identified, and 12 up-regulated and 7 down-regulated genes were detected in HIV infection and PAH (Figures 2E,F).

### 3.2 Functional enrichment analysis of shared genes between HIV infection and PAH

**Figure 3:**
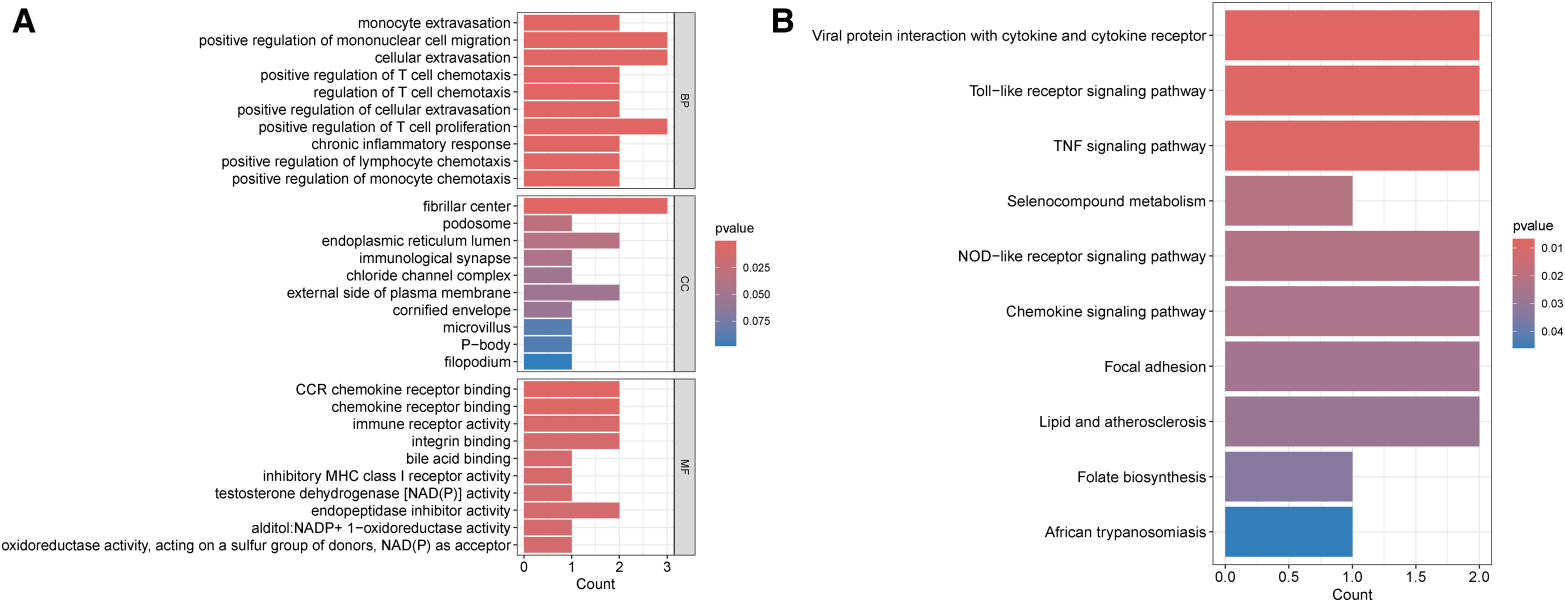
Enrichment analysis of common DEGs between HIV infection and PAH. (**A**) GO enrichment analysis of common DEGs between HIV infection and PAH. (**B**) KEGG enrichment analysis of common DEGs between HIV infection and PAH. DEGs, differentially expressed genes; HIV, human immunodeficiency virus; PAH, pulmonary arterial hypertension; GO, gene ontology; KEGG, Kyoto encyclopedia of genes and genomes; BP, biological process; CC, cellular component; MF, molecular function.

To investigate the possible common mechanism between HIV infection and PAH, GO and KEGG enrichment analyses were performed. According to GO analysis, these shared genes were mostly associated with positive regulation of mononuclear cell migration, cellular extravasation, positive regulation of T cell proliferation (biological process), fibrillar center (cellular component), and CCR chemokine receptor binding (molecular function) (Figure 3A). Besides, KEGG enrichment indicated that these genes were mainly related to viral protein interaction with cytokine and cytokine receptor (Figure 3B).

### 3.3 PPI network and identification of hub genes

**Figure 4:**
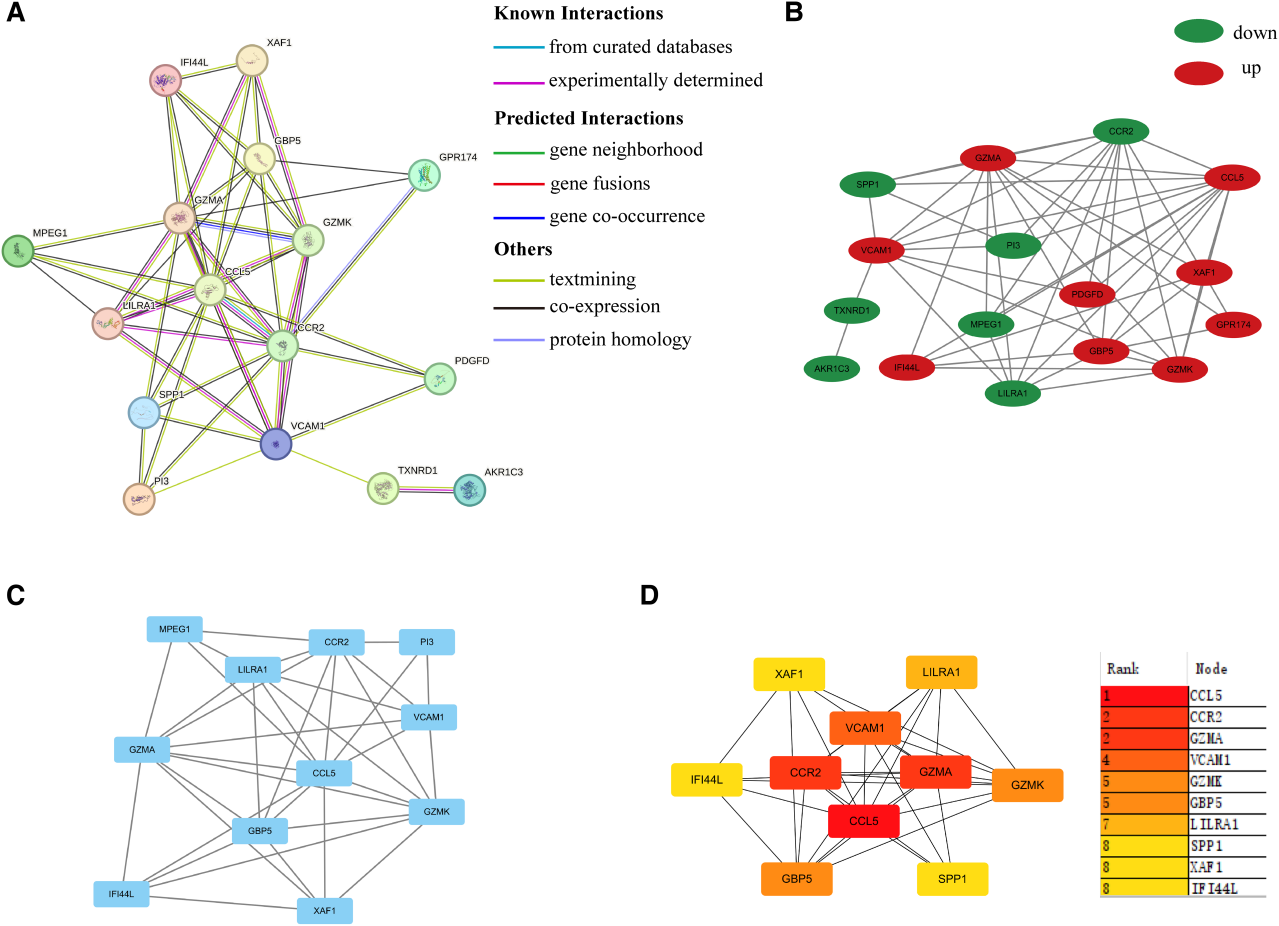
PPI network. (**A**) PPI network of common DEGs between HIV infection and PAH. (**B**) PPI network visualized by Cytoscape. Up-regulated and down-regulated genes were marked in red and green, respectively. (**C**) Sub-cluster of PPI network by MCODE. (**D**) Top ten hub genes according to Degree algorithm. PPI, protein-protein interaction; DEGs, differentially expressed genes.

As shown in Figures 4A,B, a PPI network was constructed and visualized. Next, we extracted one closely related gene cluster module using the MCODE plug-in (Figure 4C). Subsequently, the top 10 candidate hub genes were screened by the Degree algorithm. As shown in Figure 4D, the top three genes were CCL5, GZMA, and CCR2. They were closely linked.

### 3.4 Validation and diagnostic values of hub genes

**Figure 5:**
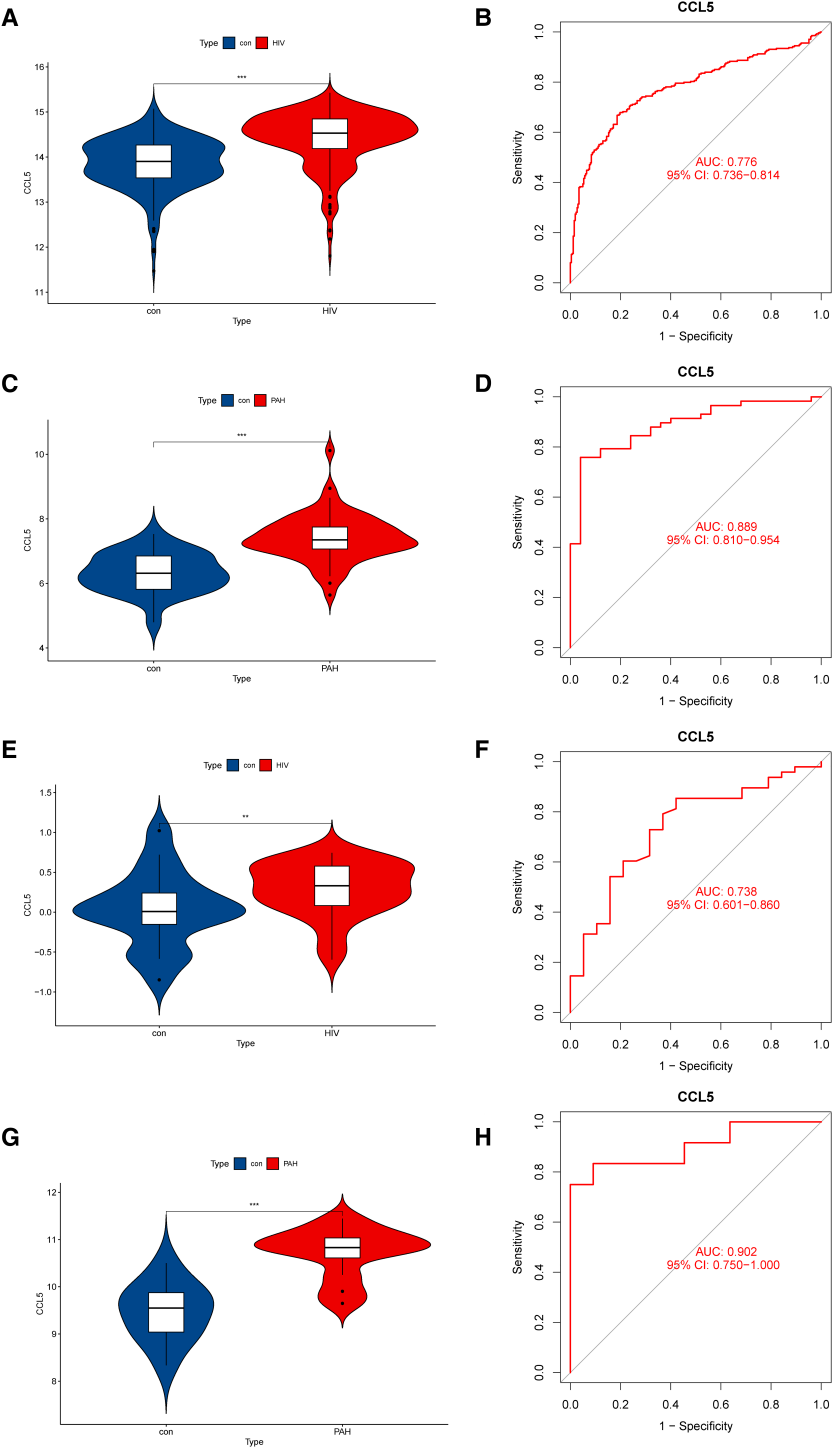
Expression levels and diagnostic values of CCL5. (**A**) Expression level of CCL5 in the discovery cohorts for HIV infection (GSE37250). (**B**) ROC curve of CCL5 in the discovery cohorts for HIV infection (GSE37250). (**C**) Expression level of CCL5 in the discovery cohorts for PAH (GSE117261). (**D**) ROC curve of CCL5 in the discovery cohorts for PAH (GSE117261). (**E**) Expression level of CCL5 in the validation cohorts for HIV infection (GSE30310). (**F**) ROC curve of CCL5 in the validation cohorts for HIV (GSE30310). (**G**) Expression level of CCL5 in the validation cohorts for PAH (GSE53408). (**H**) ROC curve of CCL5 in the validation cohorts for PAH (GSE53408). HIV, human immunodeficiency virus; PAH, pulmonary arterial hypertension; ROC, receiver operating characteristic; CCL5, CC-type chemokine ligand 5.

The expression levels of CCL5, GZMA, and CCR2 were analyzed in validation cohorts GSE30310 and GSE53408. Only CCL5 showed significant differences in both validation groups (Figures 5E,G). For dataset GSE30310, there was no significant difference in CCR2 (Supplementary Figure S2), and GZMA was not detected. Similarly, for dataset GSE53408, the expression level of GZMA was significantly increased in the PAH group (Supplementary Figure S3), but CCR2 was not detected. Therefore, CCL5 was identified as a hub gene in HIV infection and PAH.

To make our study more credible, we evaluated the expression levels and diagnostic values of CCL5 in both the discovery cohort and the validation cohort. In the discovery cohort, CCL5 was upregulated in HIV infection group (Figure 5A) and PAH group (Figure 5C). The receiver operating characteristic curves (ROC curves) showed certain diagnostic values of CCL5 (Figures 5B,D). Similarly, in the validation cohort, CCL5 was upregulated in the disease group with certain diagnostic values (Figures 5E–H).

### 3.5 Immune infiltration analysis

**Figure 6:**
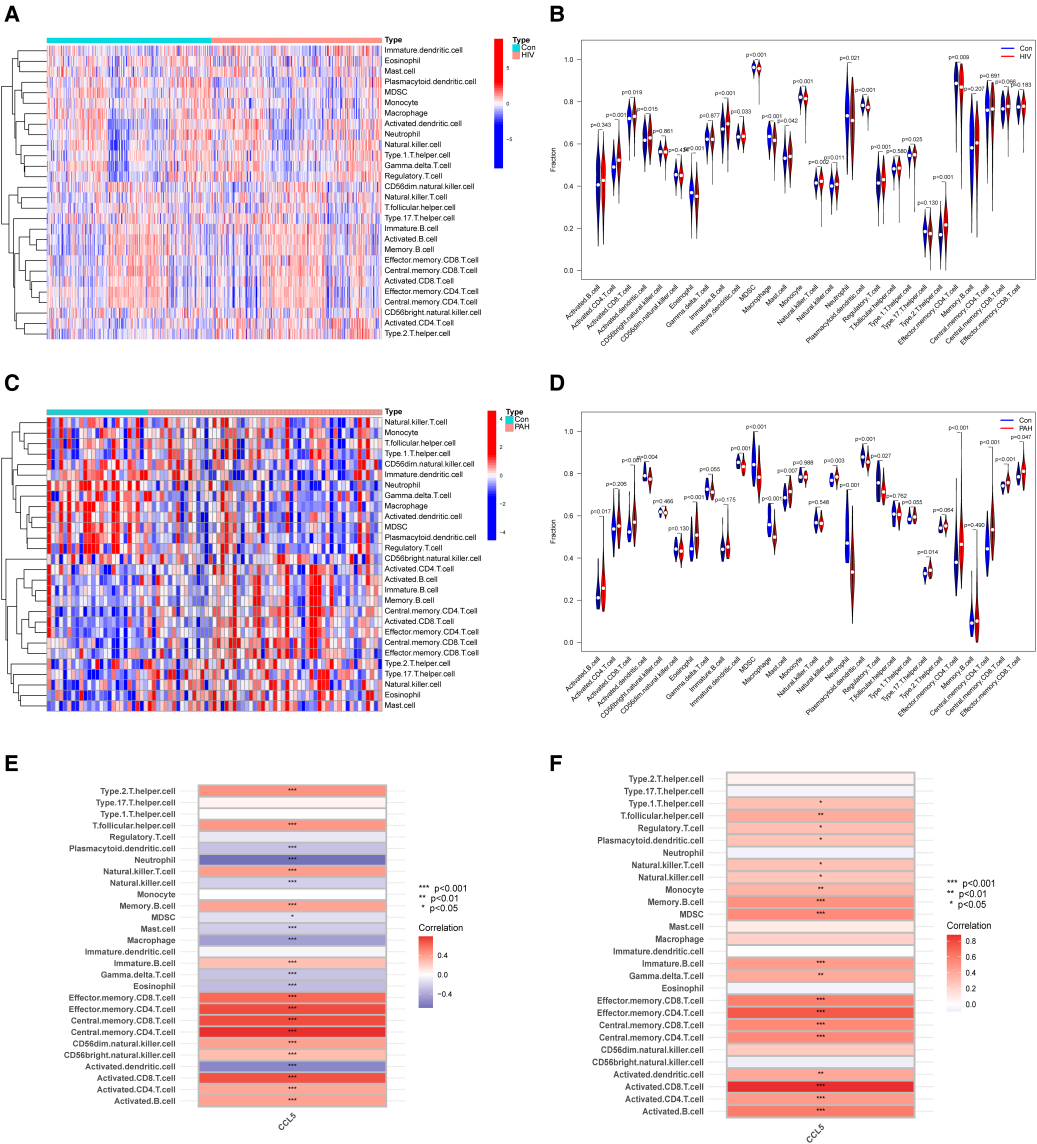
Immune infiltration analysis. (**A**) Heatmap of immune cells between control and HIV infection groups. (**B**) Violin plot of immune cells fraction between control and HIV infection groups. (**C**) Heatmap of immune cells between control and PAH groups. (**D**) Violin plot of immune cells fraction between control and PAH groups. (**E**) Correlation analysis of immune cell and CCL5 in GSE37250. (**F**) Correlation analysis of immune cell and CCL5 in GSE117261. HIV, human immunodeficiency virus; PAH, pulmonary arterial hypertension; CCL5, CC-type chemokine ligand 5.

Immune infiltration analysis showed the roles that immune cells played in HIV infection and PAH. In the HIV infection group, the proportion of activated CD4 T cells, activated CD8 T cells and activated dendritic cells increased, and there was a decline in the percentage of effector memory CD4 T cells, monocytes, and neutrophils (Figures 6A,B). In the PAH group, the proportion of activated B cells, activated CD8 T cells and eosinophils increased, and the proportion of activated dendritic cells, neutrophils, and regulatory T cells decreased (Figures 6C,D). Correlation analysis showed a correlation between CCL5 and immune cells. In dataset GSE37250, CCL5 was positively related to effector memory CD4 T cells, activated CD4 T cells, and activated CD8 T cells (Figure 6E). CCL5 was also positively correlated with the above immune cells in dataset GSE117261 (Figure 6F).

### 3.6 Validation of CCL5 expression in cells

**Figure 7:**
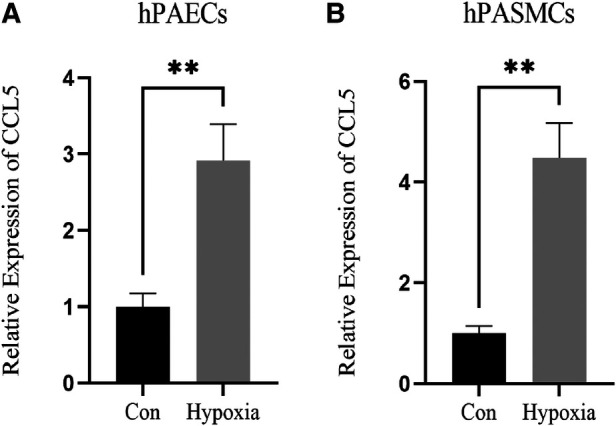
Expression of CCL5 in hPAECs and hPASMCs. (**A**) Expression level of CCL5 between control and hypoxia groups in hPAECs. (**B**) Expression level of CCL5 between control and hypoxia groups in hPASMCs. hPAECs, human pulmonary artery endothelial cells; hPASMCs, human pulmonary artery smooth muscle cells. *n* = 3, *P < 0.05, **P < 0.01.

The results of the PCR indicated that the expression of CCL5 was increased significantly in hypoxia-induced hPAECs and hPASMCs (Figures 7A,B).

### 3.7 Gene-disease association network

**Figure 8:**
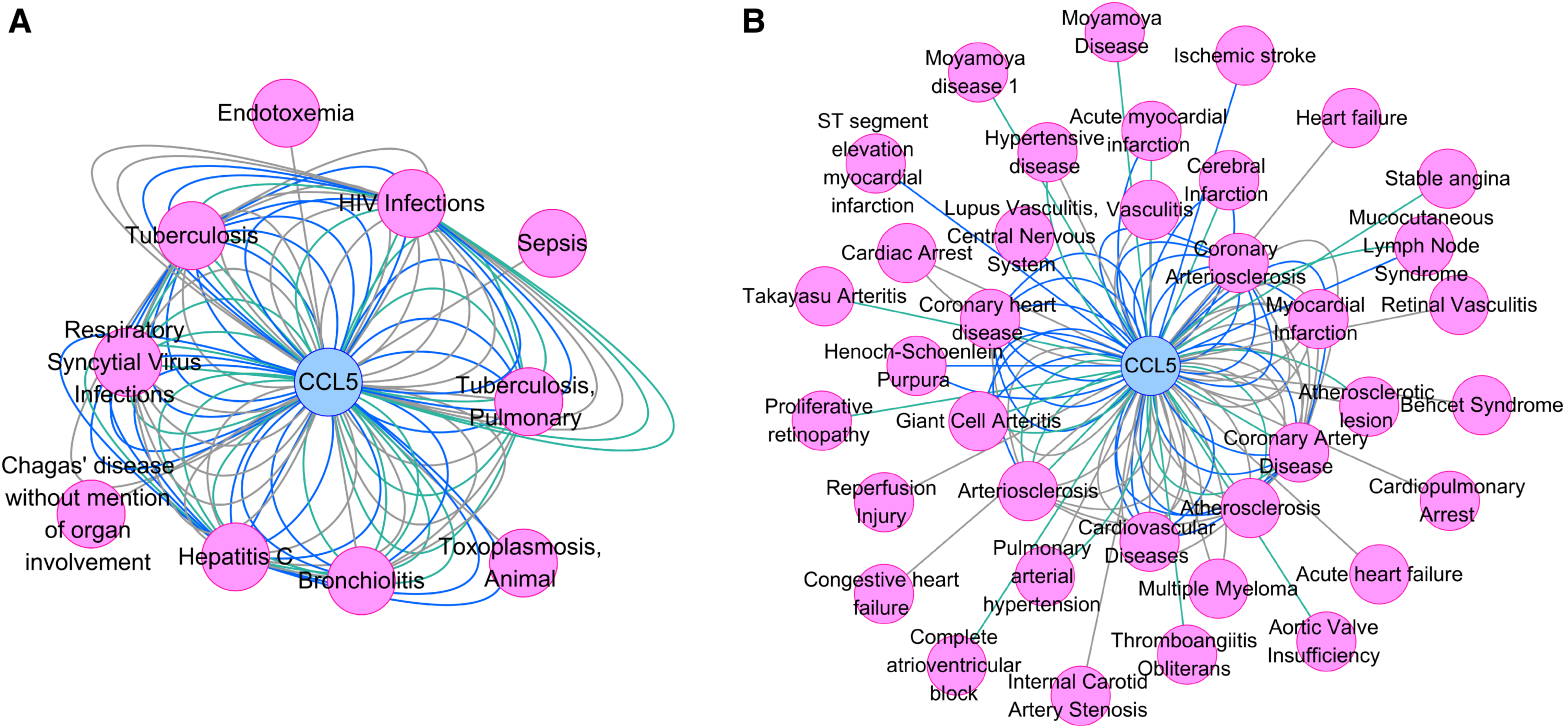
Gene-disease association network. (**A**) The association between CCL5 and infections. (**B**) The association between CCL5 and cardiovascular diseases. CCL5, CC-type chemokine ligand 5.

Figure 8A showed the association between CCL5 and infections, including HIV infection. In addition, Figure 8B showed the association between CCL5 and cardiovascular disease, including PAH.

### 3.8 Validation of docking protocol and molecular docking

**Figure 9:**
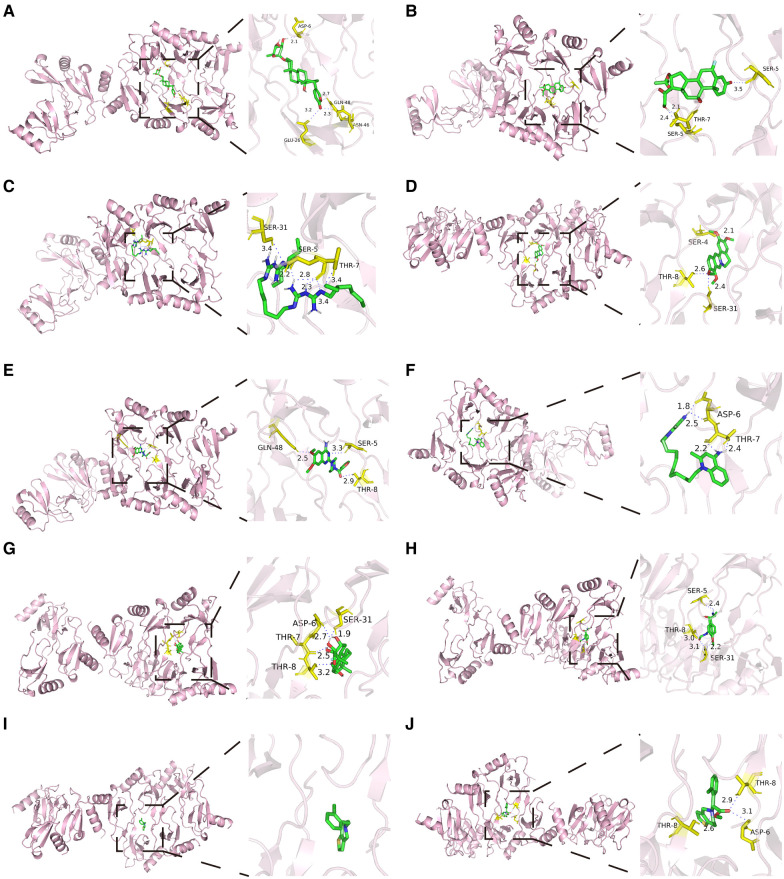
Molecular docking results of ten potential drugs and CCL5. Hydrogen bonds were displayed in blue dashed lines. Amino acid residues were labeled yellow. (**A**) Proscillaridin. (**B**) Flunisolide. (**C**) Alexidine. (**D**) Palmatine. (**E**) Prazosin. (**F**) Dequalinium. (**G**) Triamcinolone. (**H**) Formoterol. (**I**) Ticlopidine. (**J**) Clopidogrelum. CCL5, CC-type chemokine ligand 5.

The native co-crystallized ligand of CCL5 was extracted and re-docked by Autodock (Supplementary Figure S4). The calculated RMSD value was 0.266Å, which was less than 2Å, indicating that the docking protocol was valid. Besides, molecular docking was performed between the ten potential drugs and CCL5. All of the anticipated binding sites had binding energies of less than −5 kcal/mol (Supplementary Table S4), suggesting that ligands can spontaneously attach to the receptor molecule. The binding modes and binding interactions between candidate drugs (ligands) and CCL5 were displayed in Figure 9. Ligands were located in the active pocket. Except for ticlopidine, the other ligands were bound to amino acid residues around the active pocket by forming hydrogen bonds, indicating that they formed stable conformations.

## 4 Discussion

This study aimed to explore the shared biomarkers between HIV infection and PAH and seek potential therapeutic targets for HIV-associated PAH. We found that CCL5 may be a key gene in HIV-associated PAH, and several potential drugs were identified. Over the past few decades, it has been recognized that there is a correlation between HIV infection and PAH. Kim and Factor reported the first case report of a man with both HIV infection and pulmonary arterial hypertension in 1987 (34). Shortly thereafter, more similar cases were reported, which revealed a possible correlation between HIV infection and PAH (35). HIV-positive individuals have a 1,000-fold higher risk of PAH than the general population (36). So far, the pathophysiological mechanism of PAH in HIV-infected people has not been clarified, and it may be related to the HIV-viral proteins GP120, TAT, and NEF. HIV-viral proteins lead to pulmonary artery endothelial damage through inflammatory responses, which further leads to pulmonary artery remodeling and pulmonary hypertension (37). However, its mechanism at the gene level needs to be further revealed.

In this study, we performed DEGs analysis on the HIV infection dataset and the PAH dataset, respectively. By intersecting the above two DEGs sets, 19 common DEGs were obtained. Functional enrichment analyses were conducted to investigate the biological functions of the above genes. GO analysis showed that the common DEGs were mainly enriched in positive regulation of mononuclear cell migration, positive regulation of T cell proliferation, and CCR chemokine receptor binding, which are mainly involved in inflammatory responses. It is consistent with previous studies. Immune activation and chronic inflammatory responses play important roles in HIV infection. HIV targets monocyte-derived dendritic cells, monocyte-derived macrophages, and CD4 T cells with the help of chemokine receptors to activate a series of complex signaling pathways that cause the long-term activation of inflammatory responses (38, 39). Similarly, in PAH, chemokines recruit various immune cells in lung tissue, including monocytes and T cells, and promote pulmonary artery hypertension by producing a series of inflammatory factors (40). Furthermore, KEGG enrichment analysis revealed that the shared genes were mainly enriched in viral protein interaction with cytokine and cytokine receptor. This is somewhat in line with previous studies. HIV-associated PAH might possess the following pathogenesis: HIV viral proteins bind to receptors on lung endothelial cells, promoting cytokine release and inflammation, thus resulting in vascular remodeling and pulmonary hypertension (35). Therefore, we can conclude that immune activation and inflammatory responses play an important role in HIV infection and PAH.

In order to further identify the key genes, we constructed a PPI network. Moreover, it was further confirmed in the validation cohort that CCL5 was highly expressed in HIV infection and PAH, enhancing the reliability of our study.

CCL5 (CC-type chemokine ligand 5), also known as RANTES (Regulated on Activation, Normal T Cell Expressed and Secreted), is mainly secreted by activated T cells. Numerous previous investigations demonstrated that CCL5 played a crucial role in human diseases, including solid tumors (41), autoimmune diseases (42), metabolic diseases (43), etc. Similarly, CCL5 is essential for HIV infection and PAH, although the exact mechanism remains to be further studied.

CCL5 was identified as an HIV-suppressive factor produced by CD8 T cells according to a previous study (44). A clinical study showed that the expression levels of CCL5 in HIV-infected people was increased compared with HIV-negative controls (45). A meta-analysis showed that the expression level of CCL5 was negatively associated with the risk of HIV infection. CCL5 can competitively bind to CCR5 or promote the internalization of CCR5, thereby preventing the entry and replication of HIV (46). The expression level of CCL5 in individuals with HIV infection may be influenced by many factors. In HIV-positive individuals, the increased cell frequency of memory-like NK cells (47), virtual memory CD8 T cells (48), and co-infection with the human T cell lymphotropic virus (HTLV) (49) may elevate the expression of CCL5, playing a role in anti-HIV and delaying AIDS. Consistent with previous studies, we discovered that CCL5 expression was markedly elevated in the HIV infection group, which may be connected to the activation of cellular immune responses caused by viral infection.

In addition, CCL5 expression was positively correlated with the risk of PAH. A recent study showed that CCL5 expression levels were significantly elevated in the pulmonary endarterectomy tissue of patients with chronic thromboembolic pulmonary hypertension compared to healthy controls, and CCL5 may lead to pulmonary hypertension by promoting fibroblast migration (50). A study indicated that CCL5-CCR5 pathway was activated in PAH, thus promoting macrophage recruitment and pulmonary-artery smooth muscle cells proliferation (51). Besides, another study indicated that CCL5 promoted platelet activation, thus leading to endothelial cell injury and vascular remodeling in PAH (52). Furthermore, a study showed that CCL5 deficiency could reverse hypoxia-induced pulmonary hypertension by restoring bone morphogenetic protein receptor 2 (BMPR2) signaling (53). Consistent with previous studies, our study indicated that the expression level of CCL5 was increased in transcriptome data. To make our study more rigorous, *in vitro* experiments were conducted. Hypoxia-induced hPAECs and hPASMCs are commonly used to construct models of PAH. Our study indicated that CCL5 was highly expressed in hypoxia-induced hPAECs and hPASMCs, suggesting that CCL5 may promote the pathogenesis of PAH. It was consistent with our findings in the transcriptome data. In a word, although CCL5 plays a beneficial role in HIV infection, elevated levels of CCL5 promote the progression of PAH. Therefore, we can reasonably speculate that CCL5 is a key gene in HIV-associated PAH.

Regulating the expression of CCL5 may be a target for the treatment of HIV-associated PAH in the future. Previous studies have revealed several drugs that can regulate the expression of CCL5. A study showed that dimethylfumarate downregulated the secretion of CCL5 by inhibiting NF-KB (54). Similarly, Chen et al.'s study indicated that traditional Chinese medicines Shuanghuanglian and Qingkailing inhibited the expression of CCL5 by suppressing NF-kB (55). Likewise, Terminalia chebula Retz. Extract (56), desipramine, and atomoxetine (57) have been proven to downregulate the expression level of CCL5. Therefore, we speculated that the above drugs might play a role in the treatment of HIV-associated PAH by inhibiting the expression level of CCL5.

Furthermore, several drugs that target CCL5 were predicted using the DSigDB, including proscillaridin, flunisolide, alexidine, palmatine, prazosin, dequalinium, etc. Proscillaridin is a cardiac glycoside that can alleviate heart failure by inhibiting the Na+/K + pump (58), and it can also promote tumor cell apoptosis, thus playing an anti-tumor role (59). Flunisolide is a corticosteroid that is used in asthma and rhinitis by reducing inflammatory responses (60). These drugs may delay the progression of HIV-associated PAH by reducing the inflammatory responses. However, studies on the application of the above candidate drugs in HIV-related PAH are very limited. Our study provides a new idea for drug development in HIV-related PAH in the future. Due to the fact that the effect of the candidate drugs *in vivo* cannot be predicted by molecular docking, further studies are needed.

There are some advantages in our study. Firstly, previous studies on the common mechanisms of HIV infection and PAH are very limited. This was the first study to explore shared biomarkers between HIV infection and PAH by using bioinformatics methods, and the key role of inflammatory responses in HIV infection and PAH was identified by enrichment analysis and immune infiltration analysis, providing new diagnostic and treatment targets for HIV patients with PAH. Besides, the above findings were verified in validation datasets and *in vitro* experiments, which strengthened the persuasiveness of this study. Lastly, candidate drugs were predicted by molecular docking, which provided new ideas for drug development in the future.

Nevertheless, there are some shortcomings in our study. First of all, although our study was intended to explore the shared genes of HIV infection and PAH, we were unable to obtain transcriptome data from patients with both HIV infection and PAH. It is necessary to collect clinical samples for further analysis in the future. Secondly, our study did not clarify the specific mechanism by which CCL5 promotes the development of PAH, and further animal or cell experiments are required. Lastly, the specific mechanisms of interactions of 10 potential drugs with CCL5 are unknown, and further studies are required.

In conclusion, our study identified shared biomarkers in HIV infection and PAH. We illustrated that immune responses might be a key step in HIV infection and PAH, and CCL5 was a key gene in HIV-associated PAH. Finally, ten candidate drugs were predicted for HIV-associated PAH.

## Data Availability

Publicly available datasets were analyzed in this study. This data can be found here: GEO database (https://www.ncbi.nlm.nih.gov/geo/) under the accession numbers GSE37250, GSE117261, GSE30310, and GSE53408.
