# Supplementary material for: Identification and validation of CCL5 as a key gene in HIV infection and pulmonary arterial hypertension
Source: Front Cardiovasc Med. 2024 Jul 25;11:1417701. doi: 10.3389/fcvm.2024.1417701 (PMC11306045; doi:10.3389/fcvm.2024.1417701)
Supplement: Supplementary file 5 [file Datasheet1.docx]

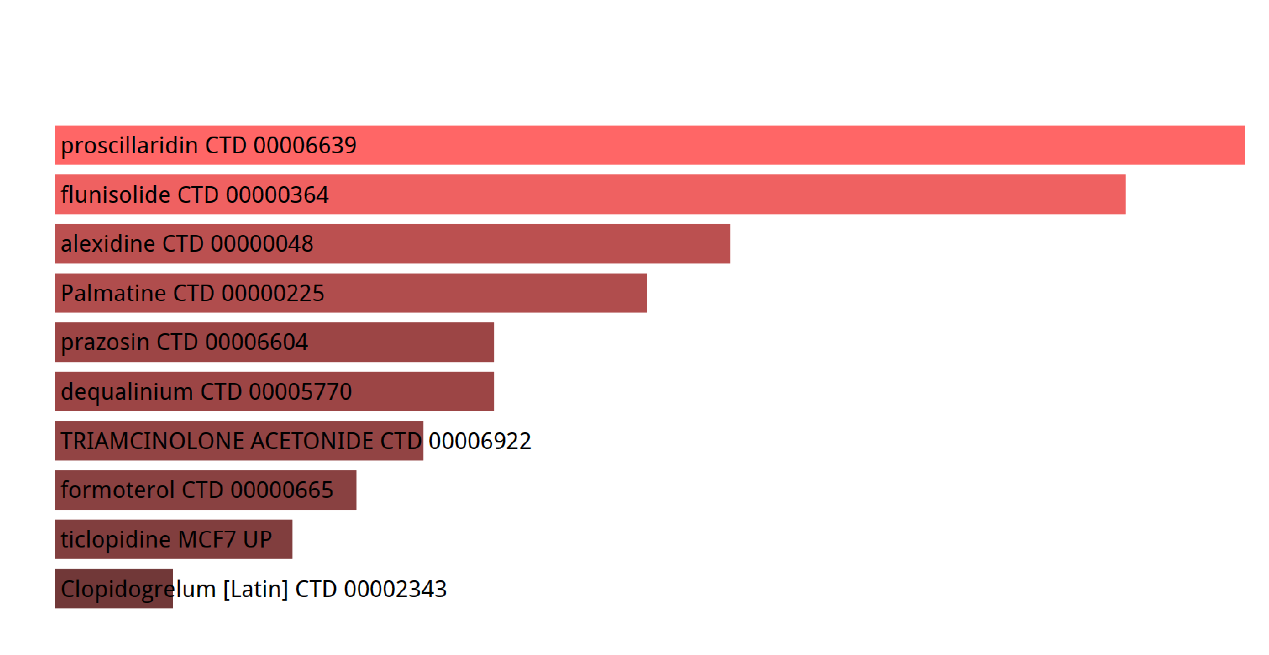


Figure S1 The top ten candidate drugs targeting CCL5 were predicted by DsigDB.


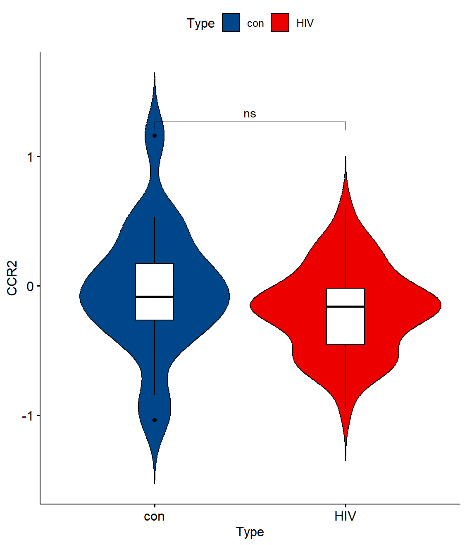


Figure S2 The expression level of CCR2 in the validation cohort GSE30310.


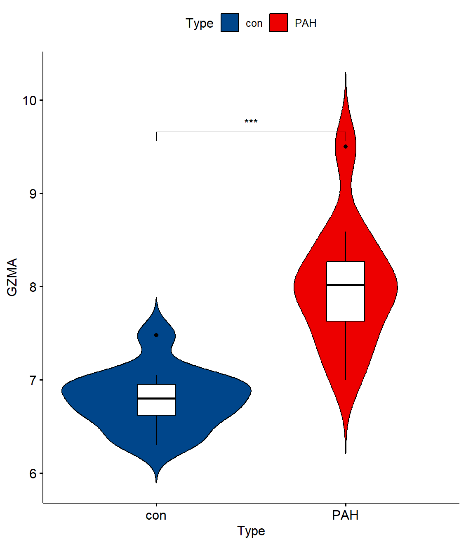


Figure S3 The expression level of GZMA in the validation cohort GSE53408.


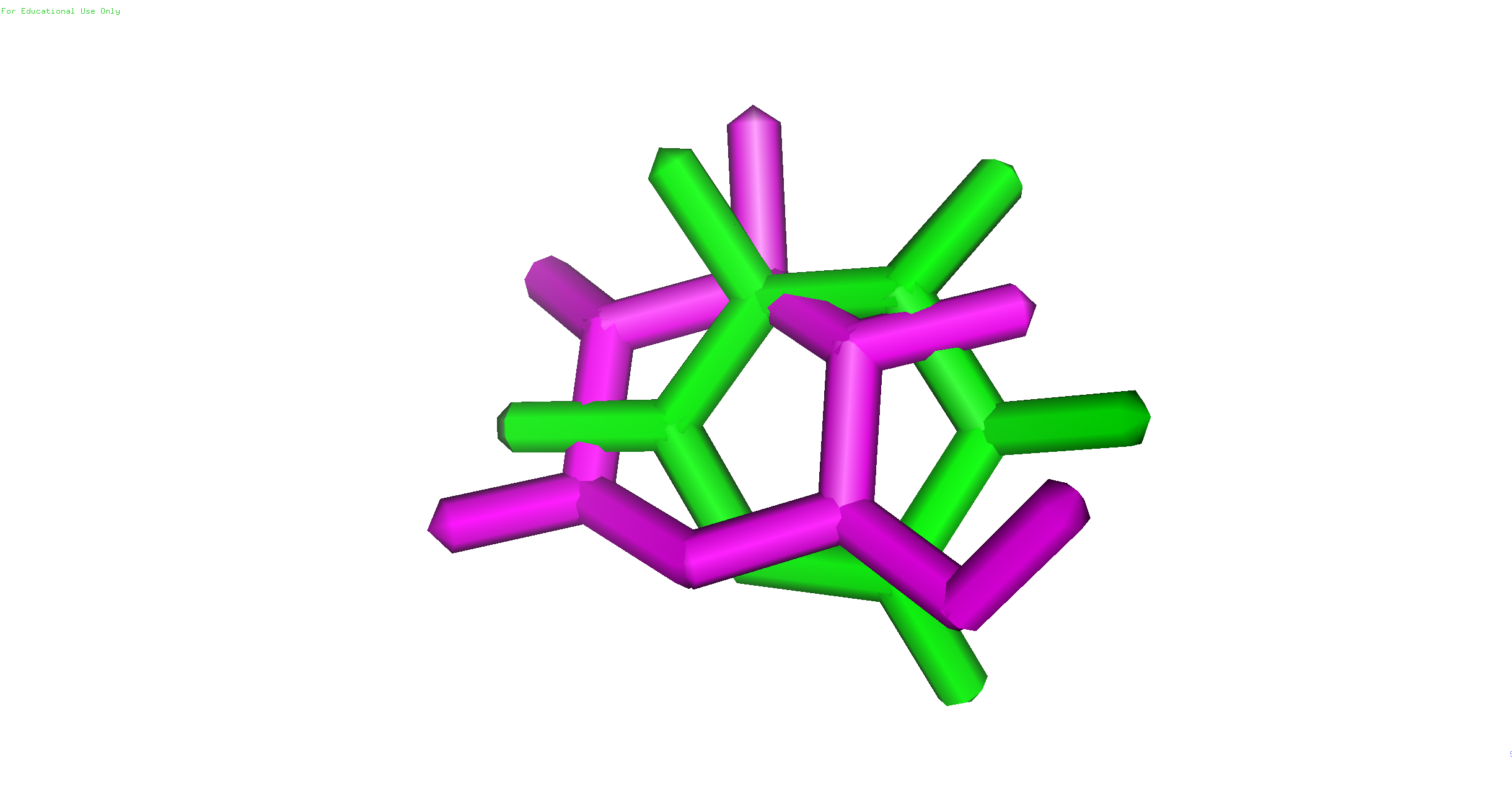


Figure S4 The co-crystalline ligand (beta-D-glucopyranose) was re-docked with CCL5. The re-docked ligand is represented in purple, whereas the original ligand is represented in green.
